# Validity of the STOP-Bang Questionnaire in Identifying OSA in a Dental Patient Cohort

**DOI:** 10.3390/medicina56070324

**Published:** 2020-06-30

**Authors:** Letizia Lonia, Marco Scalese, Gianluca Rossato, Giovanni Bruno, Francesca Zalunardo, Alberto De Stefani, Antonio Gracco

**Affiliations:** 1 Private Practice in Centro Odontostomatologico-IRCSS Sacro Cuore-Don Calabria via S. Marco 121, 37100 Verona, Italy; letizia.lonia@gmail.com; 2 Institute of Clinical Physiology, CNR, 56121 Pisa, Italy; scalese@ifc.cnr.it; 3 Neurology Department, IRCCS Sacro Cuore-Don Calabria Hospital Negrar di Valpolicella, 37024 Verona, Italy; gianluca.rossato@sacrocuore.it; 4 Department of Neuroscience, University of Padova, Via Giustiniani, 2, 35100 Padova, Italy; zalunardo.francesca@gmail.com (F.Z.); antoniogracco@gmail.com (A.G.)

**Keywords:** sleep apnea, diagnosis, OSAS, STOP-Bang questionnaire

## Abstract

*Background and objectives:* Obstructive Sleep Apnea represents a widespread problem in the population, but it is often not diagnosed and not considered a true pathology. Different diagnostic tools are available for the diagnosis of sleep apnea. This study aims to demonstrate the ability of the STOP-Bang (Snoring, Tiredness, Observed apnea, high blood Pressure, Body mass index, Age, Neck circumference, and Gender) questionnaire in identifying subjects with Obstructive Sleep Apnea (OSA) Syndrome, highlighting the role of dentists as epidemiological sentinels. *Materials and methods:* the STOP-Bang questionnaire was administered to a cohort of 1000 patients, assessing three private dental clinics in Italy. Excessive daytime sleepiness was measured using Epworth Sleepiness Scale (ESS) and defined as ≥ 10. Subjects were considered at risk of OSA if they had three or more positive items at STOP-Bang and were invited to undergo further examination with a type 3 polygraph. Presence of OSA was measured with the apnea-hypopnea index (AHI) and defined as AHI ≥ 5. *Results:* 482/1000 subjects (48.2%) had three or more positive items in the STOP-Bang questionnaire and were considered at risk for Obstructive Sleep Apnea Syndrome (OSAS). Excessive daytime sleepiness (EDS ≥ 10) was more frequent among subjects at risk for OSAS (73/482, 15.1%) vs. those not at risk for OSAS (30/518, 5.8%) (*p* < 0.0001). Moreover, 153/482 subjects at risk for OSAS (31.7%) accepted further examination with a type 3 polygraph. Presence of OSAS (AHI ≥ 5) was suggested in 121/153 subjects (79.1%, 95% CI 71.6% to 85.1%), with 76/121 subjects (62.8%) needing treatment (AHI ≥ 15). *Conclusion:* the high prevalence of OSAS highlights the role of dentists as “epidemiological sentinels”. The STOP-Bang questionnaire is a simple and efficacious instrument for screening sleep apnea patients.

## 1. Introduction

Sleep apnea is a manifestation that occurs in various pathological patterns, both respiratory and neurological. There is central apnea (of neurological interest) and obstructive apnea, typical of Obstructive Sleep Apnea (OSA).

OSA is characterized by repetitive interruption of ventilation during sleep caused by collapse of the upper (pharyngeal) airway. The obstructive apnea is a breath interruption of more than 10 s associated with ongoing ventilatory effort. Hypopnea is characterized by the decrease of ventilation without the complete arrest of it, with an associated fall in oxygen saturation or arousal. A diagnosis of Obstructive Sleep Apnea Syndrome (OSAS) is accepted when a patient has an apnea-hypopnea index (AHI; number of apneas and hypopneas per hour of sleep) >5 and symptoms of excessive daytime sleepiness, or AHI higher than 15 [1,2]. OSAS is a prevalent disorder affecting 936 million adults aged 30–69 years (men and women) regarding mild to severe obstructive sleep apnea, and 425 million adults aged 30–69 years who have moderate to severe obstructive sleep apnea [3]. It is associated with long-term sequelae, such as hypertension and cardiovascular morbidities, metabolic disorders, decrements in cognitive function, mood and quality of life, and premature death [4,5].

OSA is associated with increased risk of cardiovascular accidents and, in particular, is associated with heart attack, stroke, hypertension, and general systemic inflammatory conditions [6].

OSA-affected patients are often overweight and, in particular, obese, with Body Mass Index (BMI)’s higher than 30 kg/m^2^ [7]. The STOP-Bang (Snoring, Tiredness, Observed apnea, high blood Pressure, Body mass index, Age, Neck circumference, and Gender) questionnaire was originally designed for these patients.

Despite the important impact of morbidity and mortality, OSAS is still a poorly diagnosed disease [8,9].

The gold standard for the diagnosis of OSA is represented by nocturnal polysomnography (PSG) that can be performed in specialized structures or at patient homes [10,11,12]. However, such analyses are expensive and demanding, as they require adequate instrumentation and staff dedicated to carrying out the analyses and reporting the exams. Furthermore, patients carry out these tests only after being referred by a medical figure (typically an otolaryngologist, neurologist, or dentist), who has developed a diagnostic suspect.

Therefore, there was the urge to create a simple screening system that could be reliable, but also quick to submit to patients and easy to understand. Several screening questionnaires have been design for detecting OSA during a simple interview [13,14,15,16]. Screening tools, such as the Berlin questionnaire, STOP-Bang questionnaire, and the Epworth Sleepiness Scale (ESS) are often used for OSA, but their diagnostic accuracies are still controversial, even though the STOP-Bang Questionnaire, compared with the Berlin Questionnaire, STOP, and Epworth Sleepiness Scale, is a more accurate tool for detecting mild, moderate, and severe OSA [13].

The STOP-Bang questionnaire was first developed in 2008. It is a simple, easy to remember, and self-reportable screening tool, which includes four subjective items (STOP: Snoring, Tiredness, Observed apnea and high blood Pressure) and four demographics items (Bang: BMI, age, neck circumference, gender) [17,18,19,20].

The probability of moderate and severe OSA steadily increases with higher STOP-Bang scores. The high negative predictive value of the STOP-Bang questionnaire may indicate that patients are unlikely to have moderate-to-severe OSA [19,21].

In recent years, the importance of dentists approaching multidisciplinary problems widely increased. Dentists are often involved in the treatment of disorders that also fall within the domain of other medical specialists. Such problems include obstructive sleep apnea syndrome (OSAS).

Given the growing recognition of orofacial characteristics as important developmental factors of sleep disorders, as well as their therapeutic implications in some disorders (such as OSAS, snoring and bruxism), dentists are nowadays more conscious of sleep disorders, with a potential role in the diagnostic and therapeutic process [22,23,24,25,26].

This study investigated the ability of the STOP-Bang questionnaire in identifying subjects with OSA among patients at private dental clinics. The findings may underline the role of dentists as epidemiological sentinels, who can screen a large number of subjects and suggest further instrumental examinations (polysomnography) to achieve a diagnosis of OSA.

## 2. Materials and Methods

This prospective observational study investigated the role of the STOP-Bang questionnaire in identifying subjects with OSA, among patients accessing three private dental clinics in Veneto region (North, eastern Italy) for general dentistry control during 2014–2019.

All adult patients who accessed the private dental clinics for general dentistry control during the study period were evaluated for inclusion in the study. Patients with a previous diagnosis of OSA were excluded from the study.

The patient cohort was collected from the general population, choosing subjects at least 40 years of age, with no specific associated comorbidities. Before joining the study, patients were informed of the purpose of researching the questionnaires and completed a specific informed consent.

The sample size was calculated according to the primary aim of estimating the prevalence of OSA (defined of AHI ≥ 5) among subjects at risk, according to the STOP-Bang questionnaire (i.e., those with three or more positive items in the STOP-Bang questionnaire). Assuming a prevalence of OSA of 80% among subjects at risk, 110 subjects at risk needed to be included to estimate a 95% confidence interval not wider than 15%. The sample size was increased to 440, to take into account an expected 25% participation rate in further examination with a type 3 polygraph (i.e., 1 out of 4 subjects at risk was estimated to accept further examination with a type 3 polygraph). The sample size was further increased to 880 (according to 50% estimated prevalence of subjects at risk of OSA), which was rounded up to 1000 subjects.

Patients were asked to complete a general medical and dental anamnesis as part of a standard visit protocol, including the biometric information of height and weight for Body Mass Index (BMI) calculation. In these three private practices, the STOP-Bang (SB) questionnaire was evaluated by the same operator in every patient, according to the Italian Healthcare Guidelines for OSAS prevention.

Patients who gave three or more positive responses to the STOP-Bang questionnaire were considered at moderate or high risk of being affected by OSAS and suggested for further sleep analysis. All of the patients agreed to perform a sleep analysis with a type 3 domiciliary polygraph. The monitor used for the analysis was Embletta Multi Parameter Recorder-Polygraph (MPR-PG) (Neurolite, Bern, Switzerland). The airflow was detected with both nasal cannula and buccal thermostat. All of the polygraph reports were performed by the same neurologist expert in the sleep practice. The threshold of oxygen saturation drop was set at 3%.

Continuous data were expressed as median and interquartile range (IQR), and categorical data were expressed as frequency and percentage. Prevalence of OSAS (with 95% confidence interval) was estimated among subjects at risk. The association between excessive daytime sleepiness and risk for OSAS was evaluated with Chi Square test. All tests were two-sided and a *p*-value less than 0.05 was considered statistically significant. Data analysis was performed using R 4.0 (R Foundation for Statistical Computing, Vienna, Austria).

## 3. Results

The analysis included 1000 subjects (514 males and 486 females; median age 50 years) attending the three private clinics. Patient characteristics are shown in Table 1.

**Figure 1:**
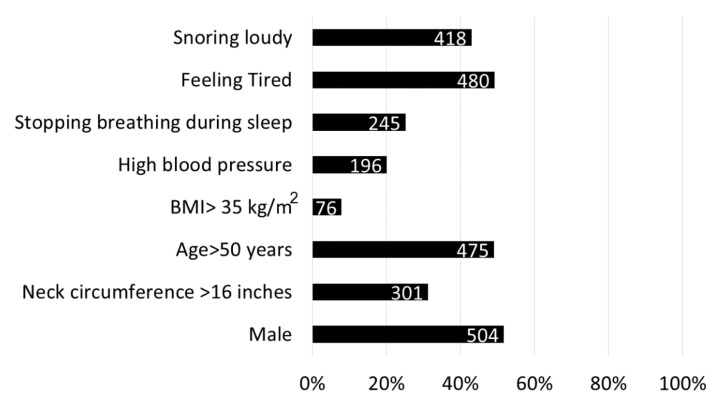
Snoring, Tiredness, Observed apnea, high blood Pressure, Body mass index, Age, Neck circumference, and Gender (STOP-Bang) items in the study sample.

The median number of positive items at the STOP-Bang questionnaire was 2 positive items (IQR 1–4). Occurrence of positive answers of each item is shown in Figure 1. IQR: interquartile range; BMI: Body Mass Index.

**Figure 2:**
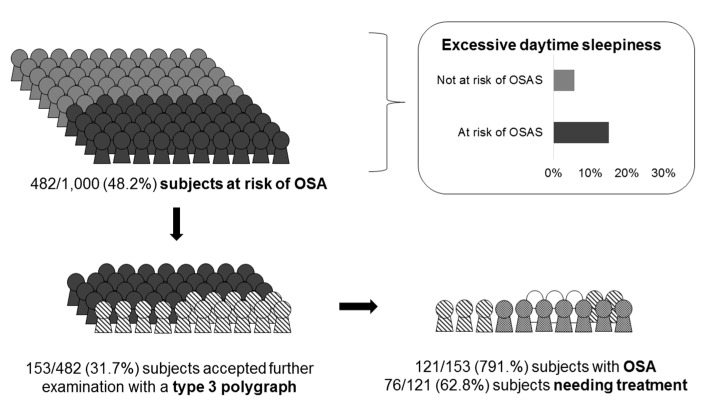
Summary of study findings. OSA: Obstructive Sleep Apnea; OSAS: Obstructive Sleep Apnea Syndrome.

Overall, 482 subjects (48.2%) had three or more positive items in the STOP-Bang questionnaire and were considered at risk for OSAS. Excessive daytime sleepiness (measured by Epworth Sleepiness Scale ≥ 10) was more frequent among subjects at risk for OSAS (73/482, 15.1%) vs. those not at risk for OSAS (30/518, 5.8%) (*p* < 0.0001). Among 482 subjects at risk for OSAS, 153 (31.7%) accepted further examination with a type 3 polygraph. Median AHI was 14 (IQR 6–29). Presence of OSAS (AHI ≥ 5) was suggested in 121 subjects (79.1%, 95% CI 71.6% to 85.1%), with 76 subjects (62.8%) needing treatment (AHI ≥ 15) (Figure 2).

## 4. Discussion

Knowing that there is a high prevalence of OSA that remains undiagnosed, it is important to stimulate clinicians from multiple medical disciplines to intercept potential patients. Indeed, OSA therapy can limit the serious cardiovascular and metabolic sequelae it entails.

The STOP-Bang questionnaire is an important tool as it does not take more than 1–2 min to complete and is easy for patients to understand [17].

Considering that most patients visit the dentist on a routine basis, the dental office represents an ideal opportunity in early disease detection and referral of patients for further evaluation [27].

Sleep specialists should use the STOP-Bang Questionnaire to conduct patient interviews for the early diagnosis of OSA in clinical settings, particularly in low-resource countries and sleep clinics where PSG is unavailable [13].

The results of this study suggest that further evaluation with polysomnography should be indicated in patients with SB ≥ 3. In fact, OSA was confirmed in 79.1% of subject at risk, with 62.8% of them requiring treatment.

The limitations of the study are related to the cohort of patients selected: the prevalence of OSA in the general population increases, in fact, starting from middle-aged men; this may have favored an increase in cases affected by the syndrome [28].

A second limitation can be linked to the voluptuous habits of the population of northern Italy: a high consumption of alcohol in the evening hours can favor the onset of night apneas [29].

A single neurologist performed the scoring of the polygraphs, without a following randomized verification: this may represent a bias in the study.

The strength of this study relies on the large cohort of included patients. Exploiting the population accessing private dental services may help in avoiding the bias associated with the investigation of patients from university clinics.

The authors’ decision to choose a cohort of patients from private dental clinics also allows us to consider the results of the study comparable to the general population.

Several researches concerning the STOP-Bang questionnaire have concentrated their cohorts of patients in specialized sleep clinics or have considered surgical patients [17].

Our findings suggest that dentists may play an important and active role in the diagnosis of OSA as diagnostic sentinel in discovering this pathology. Many patients reported that they were motivated by the dentist explaining to them the health consequences of untreated OSA. Providing specific information about OSA, and including the family members in the discussions, may increase chances of referral compliance, and are also important factors in the patient’s decision to undergo further evaluation [27,30,31].

In fact, administering the STOP-Bang questionnaire could be easily implemented in any dental practice, with the advantages of quick filling and high predictive value.

What emerges from this study is that the STOP-Bang questionnaire is an easy-to-use and valid tool for dentists who want to identify possible patients with OSA before they perform the polysomnography. This examination represents the gold standard for the diagnosis of OSA, but being very expensive from an economic and health point of view, it is not feasible for the whole population, but only for diagnostic suspects.

## 5. Conclusions

This study confirms the high frequency of OSAS and dentists roles as “epidemiological sentinels”. The STOP-Bang questionnaire (SB) is a simple and efficacious instrument for screening sleep apnea patients and it has a high predictability value on the disease.

## Figures and Tables

**Table 1:** Characteristics of the study sample.

***N* of subjects**	**1000**
**Age, years: median (IQR)**	50 (40–59)
**Sex: *n*(%)**	
**Male**	514 (51.4)
**Female**	486 (48.6)
**Weight, kg: median (IQR)**	74 (62–84)
**BMI, kg/m^2^: median (IQR)**	25.0 (22.3–28.5)
